# Drug-Free Platelets Can Act as Seeds for Aggregate Formation During Antiplatelet Therapy

**DOI:** 10.1161/ATVBAHA.115.306219

**Published:** 2015-09-23

**Authors:** Thomas Hoefer, Paul C. Armstrong, Michaela Finsterbusch, Melissa V. Chan, Nicholas S. Kirkby, Timothy D. Warner

**Affiliations:** From The William Harvey Research Institute, Barts and the London School of Medicine and Dentistry, Queen Mary University of London, Charterhouse Square, London, United Kingdom (T.H., P.C.A., M.F., M.V.C., T.D.W.); and National Heart and Lung Institute, Imperial College London, London, United Kingdom (N.S.K.).

**Keywords:** aspirin, flow cytometry, P2Y12 receptor, prasugrel, thromboxane

## Abstract

**Objective—:**

Reduced antiplatelet drug efficacy occurs in conditions of increased platelet turnover, associated with increased proportions of drug-free, that is, uninhibited, platelets. Here, we detail mechanisms by which drug-free platelets promote platelet aggregation in the face of standard antiplatelet therapy.

**Approach and Results—:**

To model standard antiplatelet therapy, platelets were treated in vitro with aspirin, the P2Y_12_ receptor blocker prasugrel active metabolite, or aspirin plus prasugrel active metabolite. Different proportions of uninhibited platelets were then introduced. Light transmission aggregometry analysis demonstrated clear positive associations between proportions of drug-free platelets and percentage platelet aggregation in response to a range of platelet agonists. Using differential platelet labeling coupled with advanced flow cytometry and confocal imaging we found aggregates formed in mixtures of aspirin-inhibited platelets together with drug-free platelets were characterized by intermingled platelet populations. This distribution is in accordance with the ability of drug-free platelets to generate thromboxane A_2_ and so drive secondary platelet activation. Conversely, aggregates formed in mixtures of prasugrel active metabolite–inhibited or aspirin plus prasugrel active metabolite–inhibited platelets together with drug-free platelets were characterized by distinct cores of drug-free platelets. This distribution is consistent with the ability of drug-free platelets to respond to the secondary activator ADP.

**Conclusions—:**

These experiments are the first to image the interactions of inhibited and uninhibited platelets in the formation of platelet aggregates. They demonstrate that a general population of platelets can contain subpopulations that respond strikingly differently to overall stimulation of the population and so act as the seed for platelet aggregation.

## Abstract 2

Supplemental Digital Content is available in the text.

Antiplatelet drugs are a cornerstone of preventive therapy for patients at risk of atherothrombotic events. Typically, these patients take low-dose aspirin (≈75–100 mg) once a day. Patients requiring further protection, such as following acute coronary syndrome with ST-segment–elevation or percutaneous coronary intervention, take a P2Y_12_ receptor blocker, most commonly clopidogrel, in addition to aspirin.^1–4^ This dosing regimen is commonly known as dual antiplatelet therapy (DAPT). Despite these therapeutic approaches, subsequent thrombotic events still occur and research has focused on ways to quantify individual patient risk. As a result, the concepts of antiplatelet drug resistance and high on-treatment platelet reactivity have developed.^5–11^ Through this research, it has become apparent that patients who have conditions associated with increased platelet turnover also have particularly elevated risks.^4,12–15^ Collectively, these observations lead to the idea that increased risk while on antiplatelet therapy may be explained by the relationship between the pharmacokinetic and pharmacodynamic properties of aspirin and P2Y_12_ inhibitors, and the dynamic changes associated with turnover of the platelet population.

**See accompanying editorial on page 2081**

Aspirin is rapidly absorbed from the stomach and small intestine reaching a plasma peak around 1 hour later.^16^ It is then rapidly metabolized by esterases in the gut, liver, and circulation and has a circulating half-life of 13 to 19 minutes. Overall, this means that little active aspirin remains in the body 2 to 3 hours after consumption. However, as aspirin irreversibly acetylates and blocks the cyclooxygenase (COX)-1 enzyme in platelets, and as anucleated platelets are limited in their ability to generate new COX-1, the inhibitory effect of aspirin on platelets persists for their entire life span of 7 to 12 days.^4,17^ The thienopyridine P2Y_12_ blockers, clopidogrel and prasugrel, are prodrugs with complex pharmacokinetics requiring metabolism to thiolactone active metabolites that are irreversible receptor antagonists of platelet P2Y_12_ receptors.^18–20^ Like aspirin, these active metabolites are short lived in the circulation but produce long-lasting effects because of irreversible inhibition of their target. Given these pharmacokinetic and pharmacodynamic profiles of aspirin and clopidogrel/prasugrel, we can reason that platelets that enter the circulation after the drugs have been cleared will not be inhibited and that the proportion of circulating drug-free platelets will increase in proportion to the rate of platelet turnover. Elevated rates of platelet production are associated with many conditions/procedures which feature increased thrombotic risk including patients undergoing coronary artery bypass surgery^21^ or those with diabetes mellitus,^22–27^ chronic kidney disease,^28,29^ metabolic syndrome,^30^ and essential thrombocythemia.^31,32^ In these patient groups, increased circulating subpopulations of uninhibited platelets may be of crucial importance in providing the seed for the formation of occlusive platelet thrombi. Drug-free platelets could also drive the changes noted in ex vivo tests of platelet reactivity.

In the in vitro study reported here, we have assessed the impact of drug-free platelet subpopulations on overall platelet responses to antiplatelet therapy by modeling the entry of drug-free platelets into the circulation and used confocal microscopy and flow cytometric imaging to provide unique images of the interactions of inhibited and drug-free platelet subpopulations in the formation of platelet aggregates. These studies provide insight into potential mechanisms underlying the disease-associated incidence of thrombotic events and illustrate important concepts relevant to ideas of antiplatelet drug resistance.

## Materials and Methods

Materials and Methods are available in the online-only Data Supplement.

## Results

### Influence of Drug-Free Platelets on Light Transmission Aggregometry Responses

**Figure 1.**
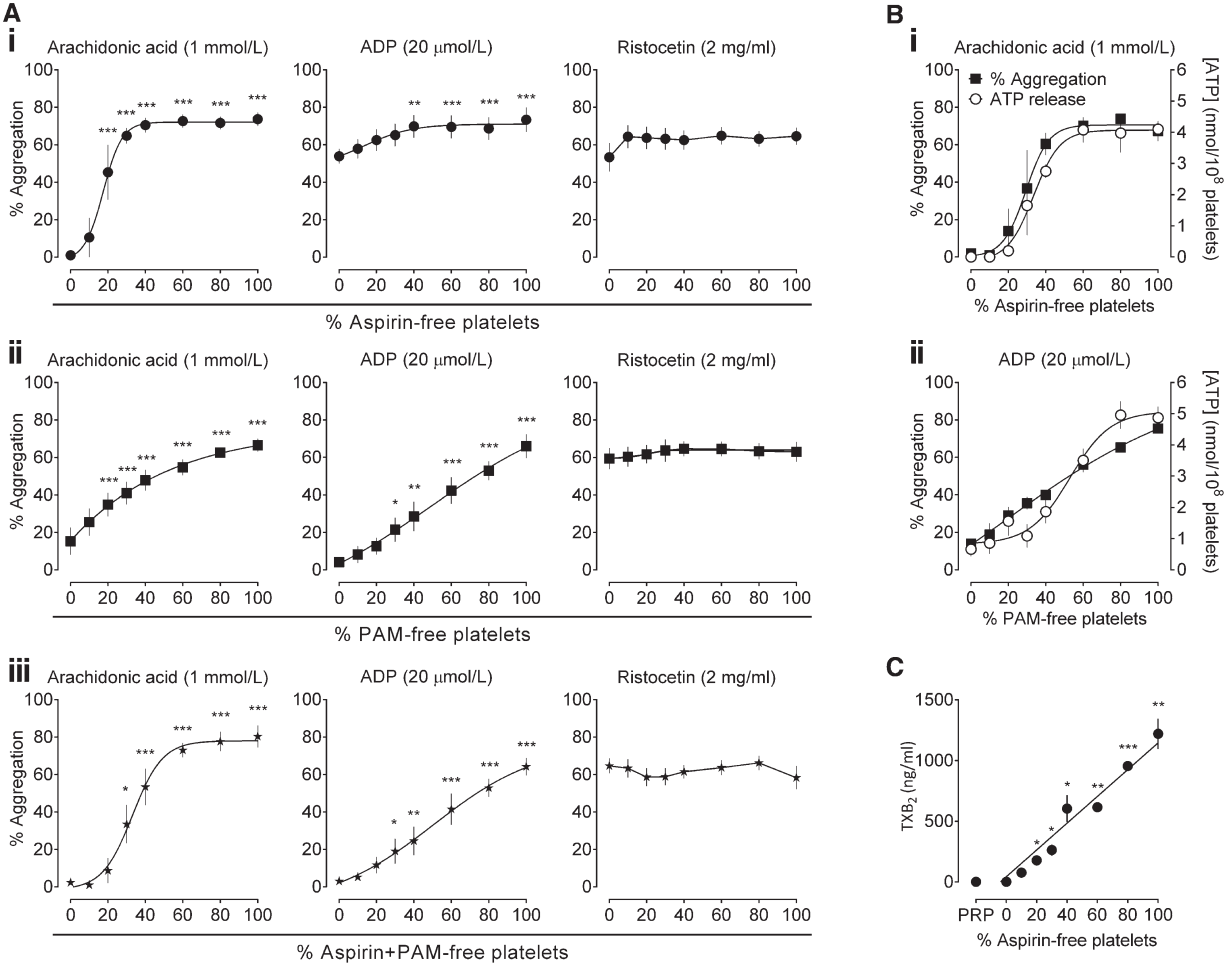
Light transmission aggregation, thromboxane A_2_ (TXA_2_) formation, and ATP-release in inhibited platelet-rich plasma (PRP) containing rising proportions of drug-free platelets. **A**, Effects of adding drug-free PRP to PRP inhibited with aspirin (30 µmol/L; **A**i), prasugrel active metabolite (PAM, 3 µmol/L; **A**ii), and aspirin+PAM (**A**iii) on aggregatory responses to arachidonic acid (AA; 1 mmol/L), ADP (20 µmol/L), or ristocetin (2 mg/mL). Curves were constructed from multiple aggregatory responses using final aggregation values obtained by traditional light transmission aggregometry (LTA) after 5-minute aggregation. Percentage aggregation values are presented as mean±SEM of experiments using platelets prepared from 6 to 8 individuals. ***P<0.001, **P<0.01, and *P<0.05 difference by paired ANOVA in aggregation from 0% drug-free platelets. **B**, Percentage aggregation and release of ATP, a measure of granule release, stimulated by AA or ADP in PRP pretreated with aspirin (30 µmol/L; **B**i) or PAM (3 µmol/L), respectively, (**B**ii) mixed with increasing proportions of drug-free PRP. Curves were constructed from multiple aggregatory responses using aggregation values obtained by traditional LTA after 5 minutes and maximum ATP released measured by CHRONOLUME assay. Percentage aggregation values and ATP-release values are presented as mean±SEM of experiments using platelets prepared from 4 individuals for each comparative point. **C**, Concentrations of TXB_2_, a measure of TXA_2_, in unstimulated PRP or rising proportions of drug-free PRP mixed with aspirin-treated PRP on stimulation by AA (1 mmol/L). Values are presented as mean±SEM of experiments using platelets prepared from 4 individuals. ***P<0.001, **P<0.01, and *P<0.05 difference by paired ANOVA in aggregation from 0% aspirin-free platelets.

First, we modeled the effects of drug-free platelets on responses recorded by light transmission aggregometry (LTA) having previously established the effectiveness of antiplatelet drugs (Table I in the online-only Data Supplement). Increasing proportions of drug-free platelets were added to inhibited platelet populations, and the net aggregatory responses were recorded (Figure 1; Figure II in the online-only Data Supplement). Aggregation to the COX-1 substrate arachidonic acid (AA; 1 mmol/L) in an aspirin-inhibited platelet population was returned to a full response by the inclusion of 30% drug-free platelets (Figure 1Ai). In general terms, increasing aggregation was seen with increasing platelet number in the absence of drug, that is, shifting from platelet-poor plasma to platelet-rich plasma (Figure III in the online-only Data Supplement). Measurement of thromboxane B_2_ (TXB_2_) as a measure of the formation of the COX product TxA_2_ demonstrated a linear increase of TXB_2_ with increasing proportions of drug-free platelets (*R*^2^=0.8867), ranging from 1.6±0.4 ng/mL TXB_2_ when 0% platelets were aspirin-free to 1220±126 ng/mL when 100% platelets were aspirin-free (Figure 1C). Similarly, aggregation to AA (1 mmol/L) in a prasugrel active metabolite (PAM)–inhibited platelet population was returned to a full response by the inclusion of 60% drug-free platelets (Figure 1Aii), and 80% drug-free platelets in the presence of an aspirin+PAM-inhibited platelet population (Figure 1Aiii). In each case, this relationship was nonlinear.

In contrast, for the P2Y_12_ receptor ligand ADP there was a linear increase in aggregation that followed the addition of drug-free platelets to platelets inhibited with either PAM or aspirin+PAM (Figure 1B and 1C). For instance, in PAM-inhibited platelets addition of 20%, 40%, and 80% drug-free platelets increased aggregation from the control level of 4±3% to 13±5%, 29±8%, and 53±5%, respectively. Conversely, the addition of drug-free platelets to PAM-treated platelets led to an inverse relationship with phosphorylation of vasodilator-stimulated phosphoprotein, a downstream measure of P2Y_12_ inhibition, in the total platelet population (Figure IV in the online-only Data Supplement). Aspirin alone had little effect on aggregation induced by ADP and so responses of aspirin-inhibited platelets were minimally affected by the addition of drug-free platelets (Figure 1Ai). Similarly, aggregation in response to U46619 was linearly related to rising proportions of drug-free platelets mixed with PAM- or aspirin+PAM-inhibited platelets, but insensitive to aspirin-treatment (Figure II in the online-only Data Supplement).

Aspirin or PAM alone partially inhibited aggregations induced by the primary stimulus collagen or the glycoprotein (GP) VI agonist collagen-related peptide, which were returned to full responses by the addition of 40% or 60% to 80% drug-free platelets, respectively. The combination of aspirin+PAM required 80% drug-free platelets for the aggregation to collagen to return to a full response and 60% for collagen-related peptide (Figure II in the online-only Data Supplement).

Similar to the first series of experiments (Table I in the online-only Data Supplement), responses to ristocetin were unaffected by aspirin (Figure 1Ai), PAM (Figure 1Aii), or aspirin+PAM (Figure 1Aiii), consistent with the mechanism of action of ristocetin being platelet agglutination rather than platelet activation, and so independent of the generation of secondary mediators of aggregation.

In inhibited platelet populations, lumiaggregometry confirmed that the increases in platelet aggregations in response to AA or ADP following addition of increasing proportions of drug-free platelets correlated with matched increases in platelet release of ATP (Figure 1B). For example, aggregations induced by AA in the presence of 10%, 40%, and 80% aspirin-free platelets were 1±1%, 60±6%, and 74±3%, whereas the accompanying releases of ATP were 0, 3±0.1, and 4±1 nmol/10^8^ platelets.

In order to mimic physiologically more relevant conditions, aggregation was also stimulated by the combination of subthreshold concentrations of the platelet agonists ADP, collagen, thrombin receptor activator peptide-6, and U46619. Aggregation in an inhibited platelet population was returned to a full response by the inclusion of 40% drug-free platelets in the presence of aspirin, 60% drug-free platelets in the presence of PAM, and 80% drug-free platelets in the presence of aspirin+PAM (Figure IID in the online-only Data Supplement).

### Aspirin-Free Platelets Intermingle With Aspirin-Inhibited Platelets, Whereas Pam-Free Platelets Form Clusters in the Center of Aggregates Covered in PAM-Inhibited Platelets

**Figure 2.**
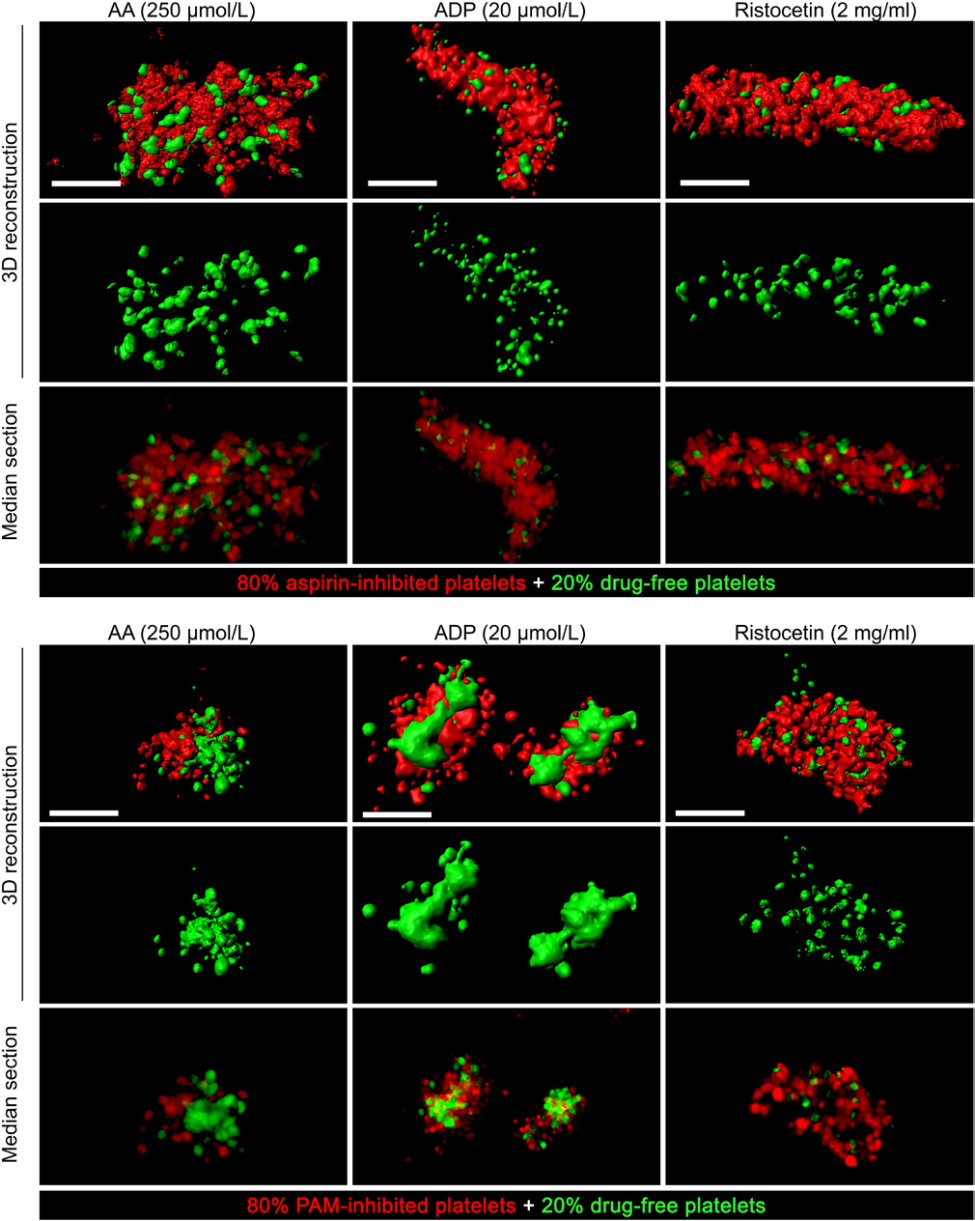
Interaction between aspirin- or prasugrel active metabolite (PAM)–inhibited platelets and a drug-free subpopulation of platelets. Confocal images of platelet aggregates formed from combined platelet populations containing 80% aspirin-inhibited (red, **A**) or PAM-inhibited (red, **B**) platelets and 20% drug-free (green) platelets. Washed platelet aggregates were obtained at the end of 5-minute light transmission aggregometry (LTA) responses stimulated by arachidonic acid (AA; 250 µmol/L), ADP (20 µmol/L), or ristocetin (2 mg/mL). For experiments, platelet suspensions were pretreated with aspirin (30 µmol/L), PAM (3 µmol/L), or corresponding vehicle for 20 minutes, washed and labeled with either PKH67 (green) or PKH26 (red) before mixing and stimulation. Images were processed with Imaris software to show images of inhibited (red) and drug-free (green) platelets. **Bottom** row of both panel sets show 5-µm median focal sections. Scale bars indicate 20 µm. Each image is representative of images from platelets prepared from at least 4 different individuals.

To elucidate the contribution of drug-free platelets to platelet aggregation, the morphologies of aggregates containing mixed platelet subpopulations were characterized by confocal imaging. Platelet aggregates comprising aspirin-inhibited platelets with a 20% subpopulation of drug-free platelets were characterized by intermingling of platelet populations (Figure 2A). Conversely, aggregates formed from combinations of 80% PAM-inhibited platelets and 20% drug-free platelets demonstrated clear clustering of drug-free platelets to the cores of platelet aggregates on stimulation by ADP or AA. Aggregates formed by stimulation of the mixed platelet populations with ristocetin were always characterized by intermingled platelet populations (Figure 2B). Analysis of confocal images revealed 5× greater clustering of drug-free platelets in the presence of PAM after ADP stimulation than the clustering after stimulation by ristocetin.

**Figure 3.**
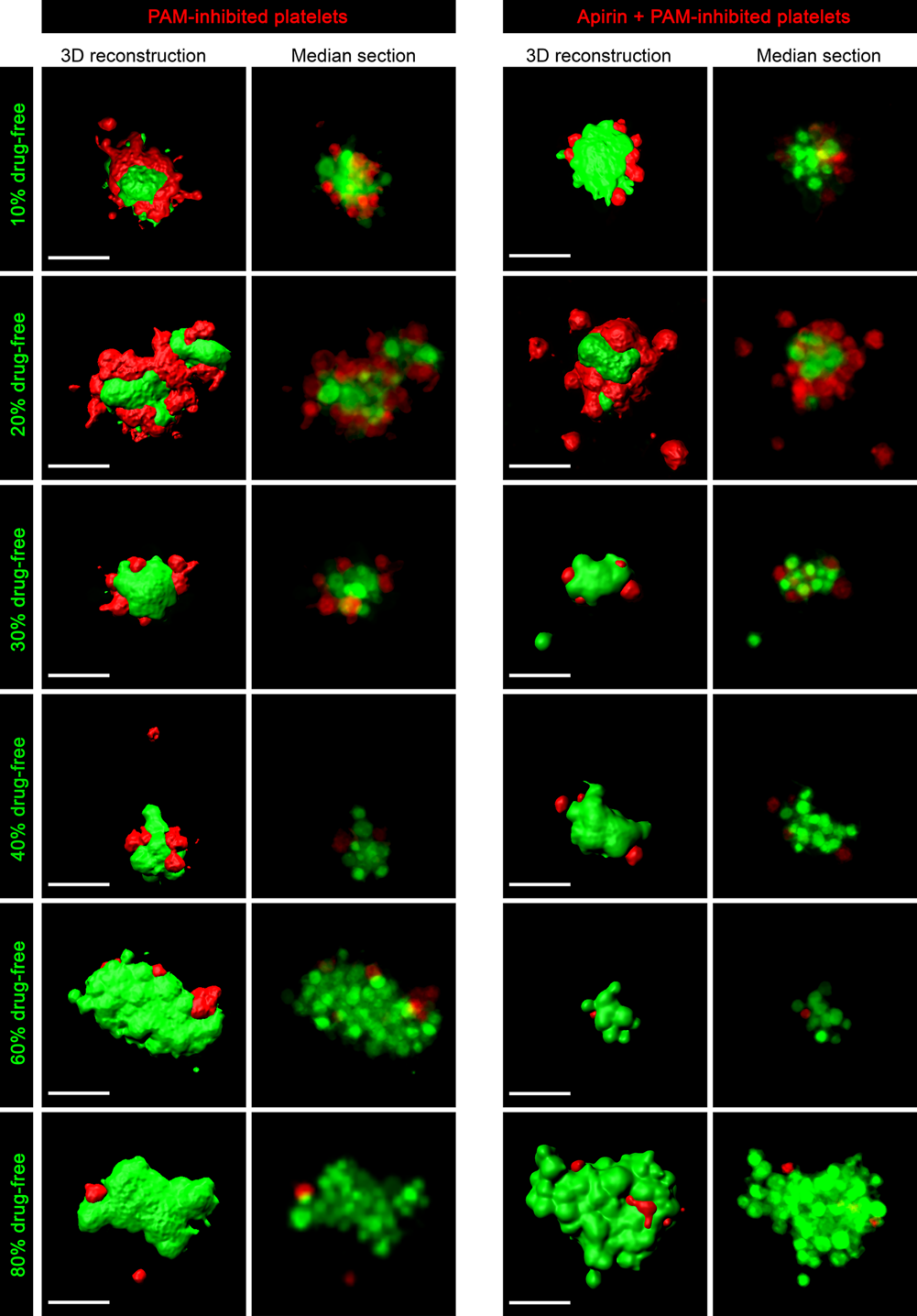
Interaction between prasugrel active metabolite (PAM) or aspirin+PAM-inhibited platelets and a drug-free subpopulation of platelets. Three-dimensional (3D) reconstruction and median sections of confocal images of platelet aggregates formed from combined platelet populations containing 90% to 20% PAM-inhibited platelets (red, **left** panels) or aspirin+PAM-inhibited platelets (red, **right** panels) and 10% to 80% drug-free platelets (green). Washed platelet aggregates were obtained at the end of 5-minute light transmission aggregometry (LTA) responses stimulated by ADP (20 µmol/L). For experiments, platelet suspensions were pretreated with aspirin (30 µmol/L) plus PAM (3 µmol/L) or corresponding vehicle for 20 minutes, washed and labeled with either PKH67 (green) or PKH26 (red) before mixing and stimulation. Images were processed with Imaris software to show combined images of inhibited (red) and drug-free (green) platelets. Scale bars indicate 10 µm.

In further studies, a full range of combinations of PAM-treated platelets or the clinically relevant combination of aspirin+PAM-treated platelets with drug-free platelets were examined. In these experiments, the central clustering of drug-free platelets in aggregates increased in proportion to the fraction of drug-free platelets (Figure 3).

### GP IIb/IIIa Mediates Recruitment of PAM-Inhibited Platelets to the Aggregate Core Independently of TXA_2_

**Figure 4.**
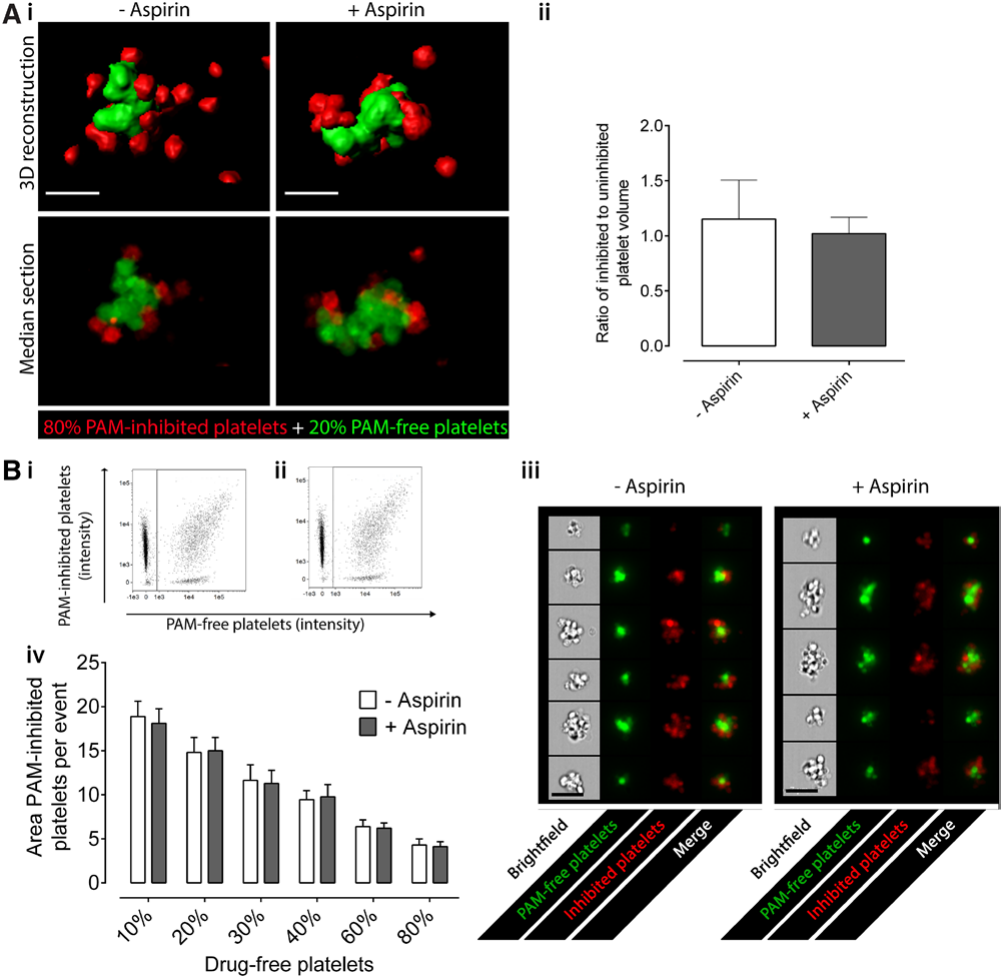
Aspirin has no effect on the association of inhibited platelets to prasugrel active metabolite (PAM)–free platelet aggregates. **A**i, Confocal images of platelet aggregates formed from combined platelet populations containing 80% PAM-inhibited or 80% aspirin+PAM-inhibited platelets (red) and 20% drug-free or aspirin-inhibited (green) platelets. Washed platelet aggregates were obtained at the end of 5-minute light transmission aggregometry (LTA) responses stimulated by ADP (20 µmol/L). For experiments, platelet suspensions were pretreated with PAM (3 µmol/L), PAM (3 µmol/L) plus aspirin (30 µmol/L), aspirin (30 µmol/L), or corresponding vehicle for 20 minutes, washed and labeled with either PKH67 (green) or PKH26 (red) before mixing and stimulation. Images were processed with Imaris software to show combined images of PAM-inhibited (red) and drug-free/aspirin-treated platelets (green). Scale bars indicate 10 µm. Each image is representative of images from platelets prepared from at least 4 different individuals. **A**ii, Six confocal images per condition were analyzed for ratios between red and green platelet volume. Volumes of platelet subpopulations were calculated by Imaris software, and different treatments were compared by *t* test and found not to be significantly different. Scatter plots of combined platelet subpopulations consisting of 20% drug-free and 80% PAM-inhibited platelets in the absence (**B**i) or presence of aspirin (**B**ii) post stimulation by ADP (20 µmol/L). **B**iii, Aggregates containing drug-free platelets were gated in region PAM-free positive and then analyzed by ImageStream. Ch1 shows the bright field image, Ch2 and Ch3 show the channel for PAM-free and PAM-inhibited platelets, respectively (scale bars indicate 14 µm). Washed platelet aggregates were obtained at the end of 5 minutes LTA responses stimulated by ADP (20 µmol/L). For experiments, platelet suspensions were pretreated with PAM (3 µmol/L), aspirin (30 µmol/L) plus PAM (3 µmol/L), aspirin (30 µmol/L), or corresponding vehicle for 20 minutes, washed and labeled with either PKH67 (green) or PKH26 (red) before mixing and stimulation. **B**iv, ImageStream data from 4 experiments were analyzed for the area of inhibited platelets associated with drug-free platelets over a range of platelet subpopulation proportions in the absence or presence of aspirin. Different treatments were compared by 2-way ANOVA and found not to be significantly different for all tested proportions.

To assess the contribution of TXA_2_ potentially formed by the drug-free core to the association of inhibited platelets to the platelet aggregates, mixed subpopulations containing drug-free and PAM-treated platelets were treated with aspirin (thereby inhibiting TXA_2_ formation) or corresponding vehicle before aggregation to ADP. Analysis of confocal images indicated no difference in the ratio of PAM-inhibited to drug-free platelet volume in formed aggregates in the absence (1.15±0.35) or presence of aspirin (1.02±0.15; Figure 4A). This was further supported by flow cytometry–based imaging analysis demonstrating no difference in measured area of PAM-inhibited platelets between different treatments over a wide range of platelet proportions (Figure 4B).

**Figure 5.**
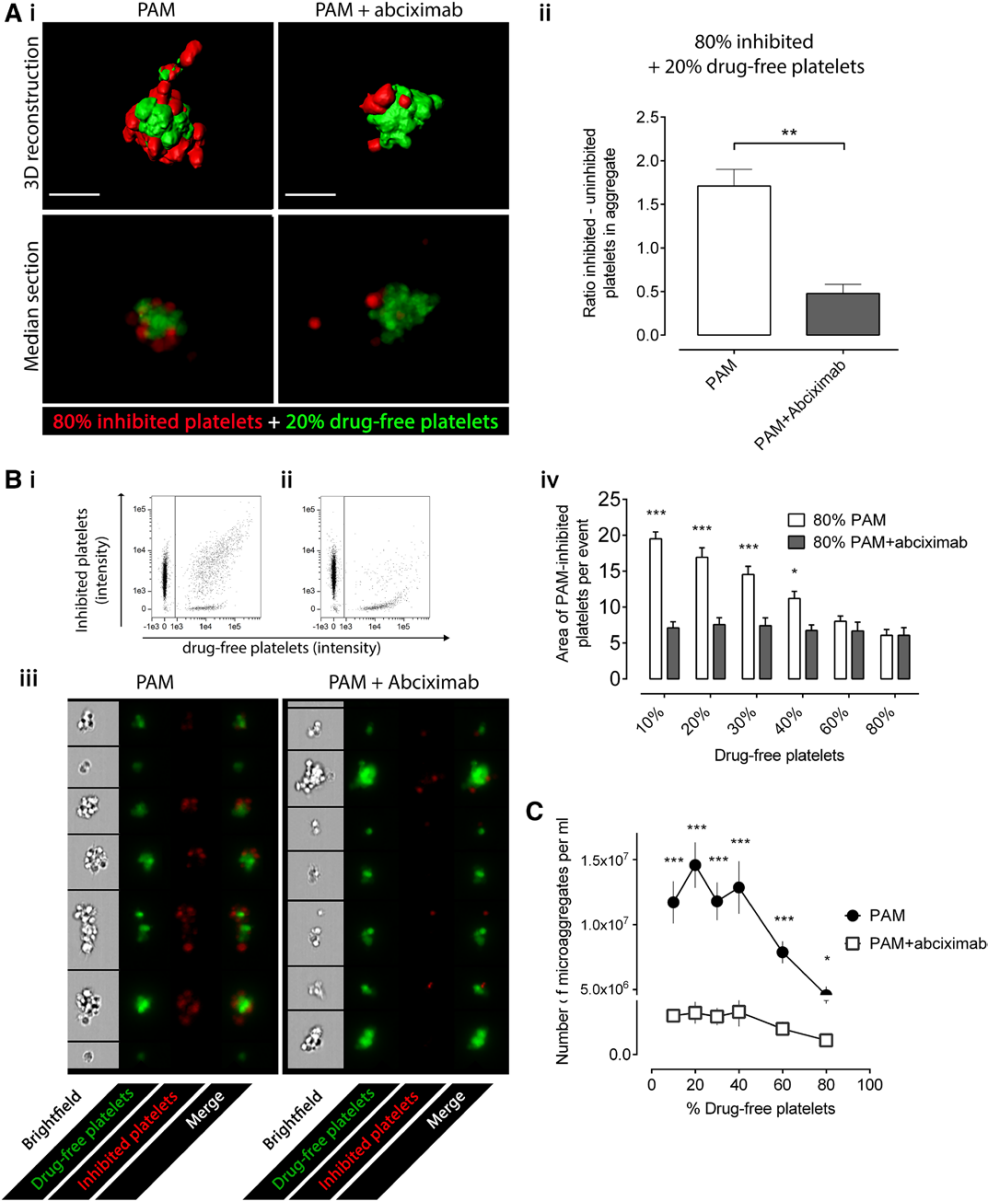
Effect of abciximab pretreatment on the interaction of drug-free and P2Y_12_-inhibited platelet subpopulations. **A**i, Confocal images of platelet aggregates formed from combined platelet populations containing 80% prasugrel active metabolite (PAM)- or PAM+abciximab-inhibited platelets (red) and 20% drug-free platelets (green). Washed platelet aggregates were obtained at the end of 5-minute light transmission aggregometry (LTA) responses stimulated by ADP (20 µmol/L). Images were processed with Imaris software. Scale bars indicate 10 µm. Each image is representative of images from platelets prepared from at least 4 different individuals. **A**ii, Six confocal images per experiment were analyzed for the ratios of red and green platelet volume. Volumes of platelet subpopulations were calculated by Imaris software and different treatments were compared by *t* test and determined as **P=0.0013 between aggregates containing PAM-inhibited platelets mixed with drug-free platelets and aggregates containing PAM+abciximab-inhibited platelets and drug-free platelets. Data represent mean±SEM of 4 experiments. Scatter plots of combined platelet subpopulations consisting of 20% drug-free and 80% PAM-inhibited platelets (**B**i) or 20% drug-free and 80% PAM+abciximab-inhibited platelets (**B**ii) post stimulation by ADP (20 µmol/L) obtained by ImageStream flow cytometry. For experiments, platelet suspensions were pretreated with PAM (3 µmol/L), PAM (3 µmol/L) plus abciximab (10 µmol/L), or corresponding vehicle for 20 minutes, washed and labeled with either PKH67 (green) or PKH26 (red) before mixing and stimulation. **B**iii, Flow cytometry–derived images of platelet aggregates formed from combined platelet populations containing 80% PAM-inhibited or PAM+abciximab-inhibited platelets (red) and 20% drug-free platelets (green; gated PAM-free positive in **B**i or **B**ii). Ch1 shows the bright field images, Ch2 and Ch3 show the channels for drug-free and PAM- or PAM+abciximab-inhibited platelets, respectively (scale bars indicate 14 µm). For analyses, washed platelet aggregates were obtained at the end of 5-minute LTA responses stimulated by ADP (20 µmol/L). **B**iv, Images obtained during flow cytometry of 4 experiments were analyzed for inhibited platelets associated with drug-free platelets over a range of platelet combinations and treatments and compared by 2-way ANOVA. ***P<0.001 and *P<0.05, n=4. **C**, Flow cytometry analysis was performed after 5-minute aggregation stimulated by ADP (20 µmol/L) in PAM- or PAM+abciximab-inhibited platelets in the presence of rising proportions of drug-free platelets. Events positive for PKH26 (inhibited platelets) and PKH67 (drug-free platelets) were identified as microaggregates. Data points represent mean±SEM of samples prepared from 5 individuals. ***P<0.001 and *P<0.05 differences of microaggregates containing drug-free and PAM-treated platelets compared with microaggregates containing drug-free and PAM+abciximab-treated platelets compared with control responses by paired ANOVA; n=6, for each.

Addition of the GP IIb/IIIa-inhibitor abciximab to PAM-inhibited platelets reduced their binding to the drug-free platelet aggregate core (0.48±0.11 versus 1.71±0.19, *P*<0.05, n=4), indicating the association of PAM-inhibited platelets to the core was not an artifact of the imaging procedure (Figure 5A).

The involvement of GP IIb/IIIa in the recruitment of PAM-inhibited platelets was confirmed by traditional flow cytometry and flow cytometric imaging analyses. Addition of abciximab to the inhibited platelet population reduced the measured platelet area of PAM-inhibited platelets associated with drug-free platelets (Figure 5B) and further caused a strong reduction in the incorporation of inhibited platelets into platelet aggregates as measured by a reduced number of aggregates containing both platelet subpopulations(Figure 5C).

**Figure 6.**
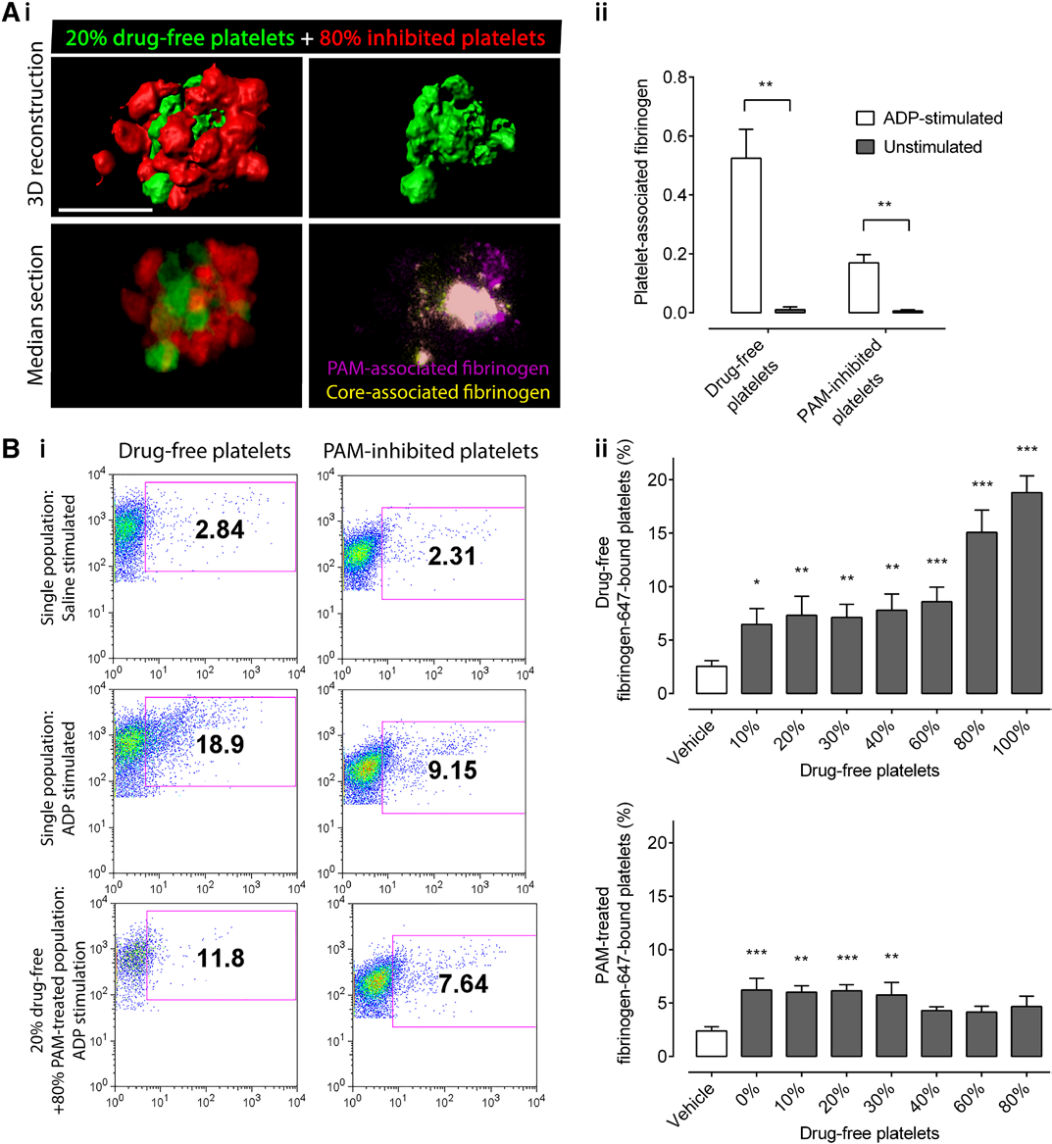
Fibrinogen is associated with both the drug-free core and the P2Y_12_-inhibited shell. **A**i, Confocal images of platelet aggregates formed from combined platelet populations containing 80% prasugrel active metabolite (PAM)–inhibited platelets (red) and 20% drug-free platelets (green) in the presence of AlexaFluor647-conjugated fibrinogen. Washed platelet aggregates were obtained at the end of 5-minute light transmission aggregometry (LTA) responses stimulated by ADP (20 µmol/L). Images were processed with Imaris software. Scale bars indicate 10 µm. Each image is representative of images from platelets prepared from at least 4 different individuals. **A**ii, Four confocal images per experiment were analyzed for the concentration of labeled fibrinogen associated with either red or green platelet volume. Volumes of platelet subpopulations were calculated with Imaris software, and fibrinogen concentrations per platelet volume were compared by *t* test and determined as *P*=0.0012 or *P*=0.002 between fibrinogen associated with unstimulated and ADP-stimulated PAM-treated platelets or drug-free platelets, respectively. **B**i, Representative flow cytometric scatter plots of drug-free or PAM-treated platelet subpopulations against AlexaFluor647-conjugated fibrinogen fluorescence (x axis) following stimulation with saline or ADP (20 µmol/L). Gated events were considered positive for fibrinogen binding and occurrence (%) calculated. **B**ii, Fibrinogen bound platelets (%) of drug-free or PAM-treated platelet populations across all tested proportions. Comparison by 1-way ANOVA found significantly higher fibrinogen binding in PAM-treated proportions compared with vehicle (saline) stimulated control. ***P<0.001, **P<0.01, and *P<0.05. Data represent mean±SEM of 4 to 5 experiments for all.

To further elucidate the underlying mechanism causing the recruitment of PAM-treated platelets into the drug-free platelet core, fibrinogen-binding studies were performed. The drug-free core was associated with the highest proportion of fibrinogen per volume of drug-free platelets (0.52±0.10) followed by the PAM-treated shell (0.17±0.03) and the control platelet sample (0.01±0.01; Figure 6A). A similar pattern was found in the 60% drug-free proportion mixed with 40% PAM-treated platelets (fibrinogen/drug-free core, 0.45±0.3; fibrinogen/PAM-treated shell, 0.1±0.06; control, 0.01±0.01; data not shown). Flow cytometry analysis of fibrinogen binding to platelet subpopulations (Figure 6B) confirmed these findings (eg, 80% PAM-treated platelets: vehicle stimulated, 2.4±0.4%; ADP-stimulated, 6.2±0.6%; *P*<0.01), indicating that GP IIb/IIIa, but not TXA_2_, is involved in the recruitment of PAM-inhibited platelets to the drug-free platelet core.

## Discussion

The confocal analyses we present here, together with quantitative data from LTA, indicate that overall aggregation responses in mixed populations of inhibited and drug-free platelets are underpinned by distinct patterns of interaction that differ between inhibition of platelet COX by aspirin and blockade of platelet P2Y_12_ receptors by thienopyridines. These findings define processes underlying in vitro platelet tests used to assess P2Y_12_ receptor blocker and aspirin effectiveness^5–11^ and provide insight to the potential interactions of platelet populations in vivo.

Others have previously reported that in tests of platelet reactivity conducted in vitro a relatively small population of aspirin naïve platelets can support full platelet aggregation, subject to the stimulus being applied. For instance, di Minno et al^15^ demonstrated that in LTA with platelet-rich plasma ≈10% drug-free platelets could support a full aggregation response to collagen (1 μg/mL) plus AA (1 mmol/L). More recent studies have associated reduced effectiveness of aspirin in vivo to increased platelet turnover as defined by the proportion of reticulated platelets in the circulation.^33^ Similar analyses have indicated that increased proportions of reticulated platelets are associated with reduced effectiveness of clopidogrel in both rats^34^ and humans,^35^ in humans receiving DAPT of aspirin plus clopidogrel,^36,37^ and most recently in humans receiving DAPT of aspirin plus prasugrel.^38^ Studies of the duration of drug action after treatment withdrawal also indicate that the return of aggregatory responses is commensurate with the time for replenishment of circulating platelets.^39^ In the first part of our studies, we modeled these effects in LTA using a panel of platelet agonists^40^ and rising proportions of drug-free platelets against a background of standard antiplatelet therapy, aspirin and aspirin plus prasugrel (by the use of PAM). We also studied PAM alone and provided aggregation curves from serially diluted platelet-rich plasma to assist in interpretation of data. Considering first the agonists most sensitive for testing the effects of aspirin, ie, AA, and PAM, ie, ADP, we noted that whereas addition of 30% of drug-free platelets could return full responses to AA in the presence of aspirin, 80% of drug-free platelets were required to return full responses to ADP in the presence of PAM or aspirin+PAM. Aspirin and P2Y_12_ receptor blockers have different targets on the platelet and inhibit with different functional modalities. Aspirin inhibits platelet COX-1 and so blocks the production of TXA_2_ by platelets. This does not stop aspirin-inhibited platelets responding to TXA_2_ produced by aspirin-free platelets, and so the hypothesis has developed that a minority of aspirin-free platelets are capable of generating sufficient TXA_2_ to support a full aggregatory response.^4,17,41^ While the in vivo proportion of aspirin uninhibited platelets required to provide a full response has been suggested to be as low as 5% or less,^41^ the response of a population of platelets to a particular concentration of TXA_2_ will depend on their existing state of activation so there is unlikely to be a particular proportion relevant in all conditions; although clearly even a small subset of platelets can produce enough TXA_2_ to aggregate a larger population.

PAM, by blocking P2Y_12_ receptors, inhibits the ability of a platelet to respond to ADP released by other activated platelets. Unlike the effects of aspirin, the blockade of one platelet’s P2Y_12_ receptors cannot be compensated for by lack of blockade on another platelet; hence, we found a linear relationship between drug-free platelets and the response to ADP. For the other platelet agonists, we noted composite responses as expected from their relative dependencies on the TXA_2_ and P2Y_12_ receptor pathways. For example, collagen and collagen-related peptide activate both pathways; U46619 is sensitive to blockade of P2Y_12_ receptors, because TXA_2_ receptor activation leads to ADP release, but not to the effects of aspirin. Consistent with this, AA stimulates the generation of TXA_2_ and so is sensitive both to aspirin, by inhibition of COX, and to PAM, because of inhibition of TXA_2_ receptor amplification.^42–44^ Overall, in the presence of aspirin+PAM, which models the effects of DAPT using aspirin and a thienopyridine, all agonists demonstrated increasing responses with increasing proportions of drug-free platelets. The exception was ristocetin, which we included as a control agent, because it causes platelet agglutination that does not require active platelet involvement.^45^

Our confocal and flow cytometric imaging studies permitted a unique and deeper exploration of the interactions between inhibited and drug-free platelet populations. Notably, drug-free platelets were distributed throughout aggregates of aspirin-inhibited platelets, whereas drug-free platelets formed clear cores within PAM- or aspirin+PAM-inhibited platelet aggregates. We conclude that the intermingled pattern of drug-free and aspirin-inhibited platelets is consistent with the drug-free subset driving the aggregatory response by producing TXA_2_ throughout the platelet mixture. In this study, we have not examined the roles of COX-1 and COX-2 isoforms because we have used mature platelet populations. However, in vivo newly formed platelets may express COX-2, which could provide a further level of complexity in the responsiveness to aspirin as could non-COX effects of aspirin.^9,31,46^ Conversely, platelets with blocked P2Y_12_ receptors cannot respond to the effects of ADP, do not become activated by ADP released as part of platelet amplification pathways, and so do not become part of platelet aggregate cores. It is interesting to contemplate that platelets with their P2Y_12_ receptors blocked are entirely mixed with drug-free platelets in our system, yet after addition of a platelet activator the drug-free platelets bind to each other so assembling an activated core from the larger mixed platelet population.

Our imaging studies primarily concentrated on the effects of a population of 20% drug-free platelets. This ratio is what could be expected under a once-daily drug regimen in conditions, such as diabetes mellitus, in which circulating platelet lifetimes are ≈5 days.^13,23,26^ For completeness, we also characterized a range of combinations between 10% drug-free and 80% drug-free platelets. These studies demonstrated in PAM-inhibited or aspirin+PAM-inhibited platelets that the size of the drug-free platelet aggregate core grew with the proportion of drug-free platelets, whereas inhibited platelets were only ever associated with the outer portion. Furthermore, we found that addition of aspirin did not reduce the association of inhibited platelets, indicating that this was not dependent on the formation of TXA_2_. However, the recruitment of P2Y_12_-inhibited platelets to the forming aggregate was the result of active aggregation because these inhibited platelets bound fibrinogen more than unstimulated control platelets, and their association to the drug-free core was reduced by the GP IIb/IIIa blocker abciximab.

**Figure 7.**
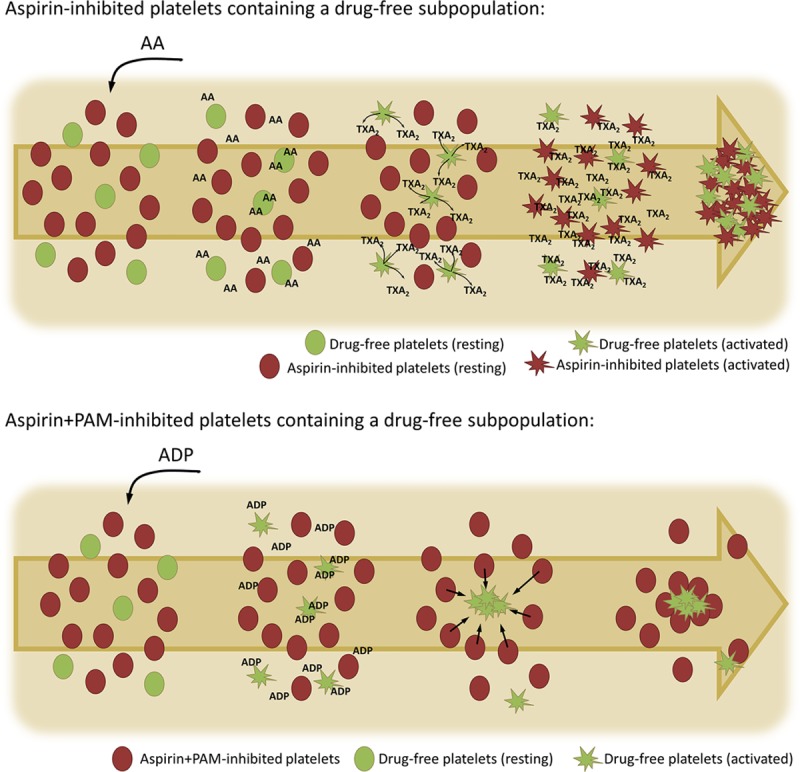
Schematic diagram of the platelet activation mechanisms resulting from presence of drug-free platelets in populations of aspirin-inhibited and dual antiplatelet-inhibited platelets. In aspirin-inhibited platelet populations mixed with drug-free platelets (**upper**), activation of cyclooxygenase-1 in drug-free platelets leads to thromboxane A_2_ (TXA_2_) formation and release. Formed TXA_2_ acts equally on both aspirin-inhibited and drug-free platelets, leading to activation and aggregate formation characterized by random intermingling of platelet subpopulations. In aspirin+PAM-inhibited platelets (modeling DAPT with an irreversible P2Y_12_ receptor blocker) combined with drug-free platelets, exposure to ADP causes activation of the drug-free subpopulation and its clustering. This leads to formation of a distinct uninhibited core that promotes activation of the inhibited platelets. In this way, through different mechanisms, drug-free platelets act as the seed for platelet aggregate formation in the presence of both aspirin and DAPT. AA indicates arachidonic acid.

In summary, our studies shed new light on the responses recorded in many studies of ex vivo platelet reactivity and their association to antiplatelet drug therapy. Although it has previously been hypothesized that subpopulations of aspirin-free platelets act as individual generators of TXA_2_, we demonstrate for the first time a mechanism by which this is achieved; aspirin-free platelets are distributed throughout aspirin-inhibited platelet aggregates allowing generated TXA_2_ to activate a larger proportion of platelets (Figure 7). In contrast, platelets free of inhibition by irreversible P2Y_12_ receptor blockers, such as clopidogrel and prasugrel, form the core of platelet aggregates and act as the nexus for the formation of larger aggregates because P2Y_12_ receptor inhibited platelets are drawn in via other activation pathways. For DAPT, this leads to a complicated interaction between the ability of a minority of aspirin uninhibited platelets to drive a full TXA_2_-dependent response and a linear relationship between P2Y_12_ receptor blockade and platelet aggregation. Aggregatory responses, measured by LTA, have been associated with thrombotic risk and these have been linked to particular patient groups in which increased platelet turnover occurs.^4,12–15^ Our in vitro studies clearly indicate that such an increased ratio of drug-free platelets may be potentially critical to modulating thrombotic risk in conditions that are associated with increased platelet turnover, such as diabetes mellitus, chronic kidney disease, metabolic syndrome, and essential thrombocythemia. This is consistent with twice a day administration of aspirin providing an improved antiplatelet effect compared with standard once a day therapy in such patient groups.^13,26,27,32,47^ In essence, our studies indicate that in a patient receiving antiplatelet therapy in the form of either aspirin or DAPT consisting of aspirin plus a thienopyridine, a drug-free subpopulation can exist that will respond differently to platelet activators. This differential interaction and its potential to drive thrombosis are important both to consideration of individualized therapies and to the development of antiplatelet strategies.

## Acknowledgments

We are grateful to Professor Sussan Nourshargh for use of confocal microscopes.

## Sources of Funding

This work was supported by grants from the Medical Research Council, the British Heart Foundation (PG-12-68-29779), the Wellcome Trust (101604/Z/13/Z), and the William Harvey Research Foundation. T.D. Warner has received research grant funding and consultancy fees from Astra Zeneca.

## Disclosures

None.

## Supplementary Material

## Significance

Many individuals at risk of heart attacks and strokes receive prophylactic antiplatelet therapy, aspirin often with the addition of a thienopyridine P2Y_12_ receptor blocker. Because of the short half lives of these drugs within the body and the continual release into the circulation of newly formed platelets, it has been hypothesized that significant populations of uninhibited platelets can emerge during standard once a day dosing. Here by modeling these events in vitro and imaging the interactions of different platelet populations, we have for the first time provided analysis of the potential underlying mechanisms and demonstrated that differently inhibited platelet subpopulations respond very differently to platelet activators. Understanding these mechanisms is central to modulating thrombotic risk in conditions that are associated with increased platelet turnover, such as diabetes mellitus, chronic kidney disease, metabolic syndrome, and essential thrombocythemia.
