# Supplementary material for: Drug-Free Platelets Can Act as Seeds for Aggregate Formation During Antiplatelet Therapy
Source: Arterioscler Thromb Vasc Biol. 2015 Sep 23;35(10):2122–33. doi: 10.1161/ATVBAHA.115.306219 (PMC4587545; doi:10.1161/ATVBAHA.115.306219)
Supplement: Supplementary file 1 [file atv-35-2122-s001.docx]

**METHODS AND MATERIALS**

**Blood collection**

Non-smoking male volunteers (aged 18-40 years) were recruited and participated in the study. Health status was determined though medical history and physical examination, including blood pressure, pulse rate, blood chemistry and urinalysis. Volunteers with normal clinical profiles were included in the study. The study was approved by St Thomas’s Hospital Research Ethics Committee (Ref. 07/Q0702/24) and all volunteers gave written consent. The volunteers abstained from aspirin, non-steroidal anti-inflammatory drugs (NSAIDs) and any other anti-platelet therapy for 14 days before ≤100 ml blood was taken from the median cubital vein using a 19G butterfly needle and collected into trisodium citrate (0.32% w/v final). The blood was immediately processed unless stated differently.

**Platelet aggregation in PRP**

Citrated blood was incubated with aspirin (30 μmol/L; A-5736, Sigma Aldrich, UK) and/or prasugrel active metabolite (PAM, 3 μmol/L; gift from AstraZeneca, Sweden) and/or vehicle for four hours. We have previously established these concentrations of aspirin and PAM to be effective and equivalent to these seen following standard oral therapy^[1-4](#_ENREF_1)^. Platelet-rich plasma (PRP) was then obtained by centrifugation (175 x *g*, 15 min, unless otherwise stated). Aggregations of different combinations of vehicle and drug-treated PRP in response to single agonists, arachidonic acid (AA, 1 mmol/L; A8798, Sigma Aldrich, UK), ADP (20 μmol/L; 3000384, Labmedics, UK), U46619 (1 μmol/L; BML-PG023-0010, Enzo, UK), collagen related peptide (CRP-XL; 0.1 μg/ml; gift from Prof Richard Farndale, University of Cambridge, UK), Horm collagen (1 μg/ml; 1130630, Takeda, Austria), ristocetin (2 mg/ml; 5372, Helena Bioscience, UK), or to the combination of the four agonists ADP (0.1 μmol/L), collagen (0.1 μg/ml), U46619 (0.1 μmol/L) and TRAP-6 (0.1 μmol/L, H29360005, Bachem, UK) were then measured in a Bio/Data PAP-8E turbidometric aggregometer using the principle of light transmission aggregometry (LTA)[^3^](#_ENREF_3)^,^[^5^](#_ENREF_5)^,^[^6^](#_ENREF_6). The final values after five minutes aggregation were recorded.

In other experiments, citrated blood from healthy volunteers was centrifuged at 175, 230, 320, 420, 610, 830 and 12000 x g in order to obtain 100, 90, 80, 70, 60, 40, 20 and 0 % total platelets, respectively. Aggregations of these different proportional platelet populations were assessed using LTA after stimulation by AA (1 mmol/L) or ADP (20 μmol/L).

Lumiaggregometry in response to AA (1 mmol/L) or ADP (20 μmol/L) was performed for 5 min in a Chronolog 560CA lumiaggregometer connected to a PC running Chart v4.2 (ADInstruments, UK). Final aggregation was recorded using the principle of LTA whereas maximum ATP release was measured by the luminometric CHRONOLUME (300395, Labmedics, UK) assay according to manufacturer’s instructions. Data was analysed using LabChart 7 reader (ADInstruments, UK).

**Aggregates for confocal microscopy**

Platelets were isolated from PRP by further centrifugation (1100 x *g*, 10 min) in the presence of prostaglandin I_2_ (PGI_2_;1 μg/ml; 2989, Tocris, UK). The resulting pellets were washed in modified Tyrode’s buffer (pH 7.4) containing HEPES (20 mmol/L; H0887, Sigma Aldrich, UK) and 0.02 U/ml apyrase (A7646, Sigma Aldrich, UK) and re-suspended in an equal volume in modified Tyrode’s buffer and incubated with aspirin (30 μmol/L), PAM (3 μmol/L), aspirin+PAM, the GPIIb/IIIa blocker, abciximab (10 μg/ml; VL4170, Eli Lilly, USA), or corresponding vehicle for 20 min at room temperature. Platelet suspensions were then centrifuged (1100 x *g*, 10 min) in the presence of PGI_2_ (1 μg/ml) again and resuspended in Diluent C (CGLDIL, Sigma Aldrich, UK) containing the fluorescent cell linker kits PKH26 or PKH67 (both 2 μmol/L; PKH26GL, PKH67GL, Sigma Aldrich, UK) which incorporate a green (PKH67) or yellow-orange fluorescent dye with long aliphatic tails (PKH26) into lipid regions of the platelet membrane[^7^](#_ENREF_7). Labelled platelets were then centrifuged (1100 x *g*, 10 min) again in the presence of PGI_2_ (1 μg/ml) and re-suspended in modified Tyrode’s buffer (containing 0.35% w/v bovine serum albumin) to a concentration of 3x10^8^ platelets/ml. Differently labelled platelet populations were then combined in various proportions and platelet aggregation measured using LTA, as above with the exception that aggregation for AA was stimulated by 250 µmol/L.

**Confocal microscopy**

Aggregates of labelled washed platelets obtained by LTA were fixed by addition of 2% formalin (HT-501320, Sigma Aldrich, UK) to the cuvettes after the aggregation. Platelet aggregates were then transferred to a microscope slide, mixed with hard set mounting medium (VECTASHIELD HardSet Mounting Medium, H-1400, Vector Laboratories, UK) and covered with a coverslip. Platelet aggregates were then imaged using a Zeiss LSM 5 PASCAL confocal laser-scanning microscope incorporating a 63 x oil-dipping Plan-APOCHROMAT objective (numerical aperture 1.4 and resolution 0.28 µm). Z-stack images were captured using the multiple track scanning mode. Volume ratios of platelet complexes were assessed from the captured images using the image processing software Imaris (Bitplane AG, Switzerland), rendering surfaces around captured fluorescence, (Figure SI). Binding of labelled fibrinogen to platelet sub-populations was calculated by application of masks onto fibrinogen-derived fluorescent signal.

**Flow Cytometry based imaging**

Aggregates of labelled washed platelets obtained by LTA were fixed by addition of 2% formalin to the cuvettes after the aggregation and transferred into a 1.5ml Eppendorf tube. Images were acquired on an ImageStream^X^ MkII imaging flow cytometer (Amnis Corp, WA, USA) incorporating a 60x objective (numerical aperture 0.9 and resolution 0.3 µm) using ISX acquisition software (Amnis Corp, WA, USA). Ideas software (Amnis Corp, WA, USA) was used for post-acquisition analysis.

**Flow Cytometry**

For quantification of micro-aggregates, defined as events positive for both PKH67 and PKH26-labelled platelets, a flow cytometric assay was used. Pre-labelled platelets were stimulated for 5 min in a light transmission aggregometer prior to fixation with 1.5% formalin. 10 µl CountBright enumeration beads (C36950, Life Technologies, UK) were added at a concentration of 1000 beads per µl. Samples were acquired on a BD FACSCalibur flow cytometer using BD CellQuest acquisition software. For quantification of fibrinogen binding by drug-free or PAM-treated subpopulations, pre-labelled platelets were stimulated for 2 min in presence of AlexaFluor647-conjugated fibrinogen (6 µg/ml, F35200, Life Technologies, UK) prior to fixation with 1.5% formalin. Samples were then diluted with saline and 15,000 platelet events acquired per sample on a BD LSRFortessa cell analyser using BD FACSDIVA acquisition software.

Vasodilator stimulated phosphoprotein (VASP) phosphorylation was quantified for different combinations of vehicle and PAM (3 μmol/L)-treated PRP stimulated by ADP (20 μmol/L) with gentle mixing. After 4 min, the reaction was stopped with methanol-free formaldehyde (2% final; PN28906, Fisher Scientific). Platelets were permeabilised (0.2% Triton X-100; 93443, Sigma) and incubated with anti–VASP-P(Ser^239^) primary antibody (ALX-804-240-C100, Enzo Life-sciences, Exeter, UK), Alexa647-conjugated secondary antibody (A21237, Invitrogen, Paisley, UK), and FITC-conjugated anti-CD42b (11-0429-71, eBioscience, Hatfield, UK), for 30 min each, in turn, before the platelet pellet was resuspended in 0.9% saline. VASP-P(Ser^239^) immunoreactivity was measured by flow cytometry with 10,000 platelet events acquired per sample using a BD FACS-Calibur instrument.

FlowJo software (Tree Star Inc, OR, USA) was used for post-acquisition analysis.

**Thromboxane measurement**

After the aggregation of drug-free PRP mixed with aspirin-treated PRP in response to AA (1 mmol/L), cyclo-oxygenase activity was arrested by the addition of diclofenac (1 mmol/L, D6899, Sigma Aldrich, UK). Platelet suspensions were then removed from the aggregometer cuvettes and the supernatant separated by centrifugation, removed and frozen at -80^°^C. The levels of TxA_2_ production were determined by measurement of its stable breakdown product, TxB_2_, using selective, competitive EIA (CAY519031, Cayman Chemical, MI, USA) in accordance with the package insert.

**Statistical Analysis**

GraphPad Prism (GraphPad Software Inc, CA, USA) was used for data analysis. Data are shown as mean±SEM. Aggregatory responses with rising proportions of drug-free platelets were analysed by one-way ANOVA, or one sample t-test for normalized data. T-tests were used to evaluate the effect of addition of abciximab to PAM-treatment, the effect of aspirin on the recruitment of PAM-inhibited platelets observed in confocal-microscopy. One-way ANOVAs or two-way ANOVAs, as appropriate, were used to evaluate the effects of different treatments (PAM vs PAM+abciximab, recruitment of PAM-inhibited platelet in the presence or absence of aspirin) over a range of platelet proportions in flow cytometry or flow cytometry based imaging. Probability values were two-tailed; p<0.05 was considered significant.

**References**

1. Leadbeater PD, Kirkby NS, Thomas S, et al. Aspirin has little additional anti-platelet effect in healthy volunteers receiving prasugrel. *Journal of thrombosis and haemostasis : JTH*. 2011;9(10):2050-2056.

2. Kirkby NS, Leadbeater PD, Chan MV, Nylander S, Mitchell JA, Warner TD. Antiplatelet effects of aspirin vary with level of P2Y(1)(2) receptor blockade supplied by either ticagrelor or prasugrel. *Journal of thrombosis and haemostasis : JTH*. 2011;9(10):2103-2105.

3. Armstrong PC, Leadbeater PD, Chan MV, et al. In the presence of strong P2Y12 receptor blockade, aspirin provides little additional inhibition of platelet aggregation. *Journal of thrombosis and haemostasis : JTH*. 2011;9(3):552-561.

4. Armstrong PC, Truss NJ, Ali FY, et al. Aspirin and the in vitro linear relationship between thromboxane A2-mediated platelet aggregation and platelet production of thromboxane A2. *J Thromb Haemost*. 2008;6(11):1933-1943.

5. Kirkby NS, Lundberg MH, Chan MV, et al. Blockade of the purinergic P2Y12 receptor greatly increases the platelet inhibitory actions of nitric oxide. *Proc Natl Acad Sci U S A*. 2013;110(39):15782-15787.

6. Cattaneo M, Cerletti C, Harrison P, et al. Recommendations for the Standardization of Light Transmission Aggregometry: A Consensus of the Working Party from the Platelet Physiology Subcommittee of SSC/ISTH. *J Thromb Haemost*. 2013.

7. Wallace PK, Tario JD, Jr., Fisher JL, Wallace SS, Ernstoff MS, Muirhead KA. Tracking antigen-driven responses by flow cytometry: monitoring proliferation by dye dilution. *Cytometry A*. 2008;73(11):1019-1034.
