# Supplementary material for: Drug-Free Platelets Can Act as Seeds for Aggregate Formation During Antiplatelet Therapy
Source: Arterioscler Thromb Vasc Biol. 2015 Sep 23;35(10):2122–33. doi: 10.1161/ATVBAHA.115.306219 (PMC4587545; doi:10.1161/ATVBAHA.115.306219)
Supplement: Supplementary file 2 [file atv-35-2122-s002.pdf]

SUPPLEMENTAL MATERIAL

TABLES

aggregatory stimulus	% of control aggregation (vehicle-treated)		
	aspirin	PAM	aspirin + PAM
AA	2±2*	27±12*	4±2*
ADP	76±6	7±4*	5±2*
collagen	22±5*	56±15*	9±2*
CRP-XL	53±10*	47±10*	37±10*
ristocetin	85±8	95±7	115±9
U46619	113±5	25±11*	24±7*

Table SI. *In vitro* effects of aspirin, PAM and aspirin+PAM on aggregatory responses of platelets. Percentage values of control vs aspirin (30 µmol/L), PAM (3 µmol/L) and aspirin+PAM aggregation in response to stimulation by AA (1 mmol/L), ADP (20 µmol/L), collagen (1 µg/ml), CRP-XL (0.1 µg/ml), ristocetin (2 mg/ml) and U46619 (1 µmol/L). are presented as mean±SEM of experiments using platelets prepared from 6 individuals; * $p<0.05$ compared to control responses.

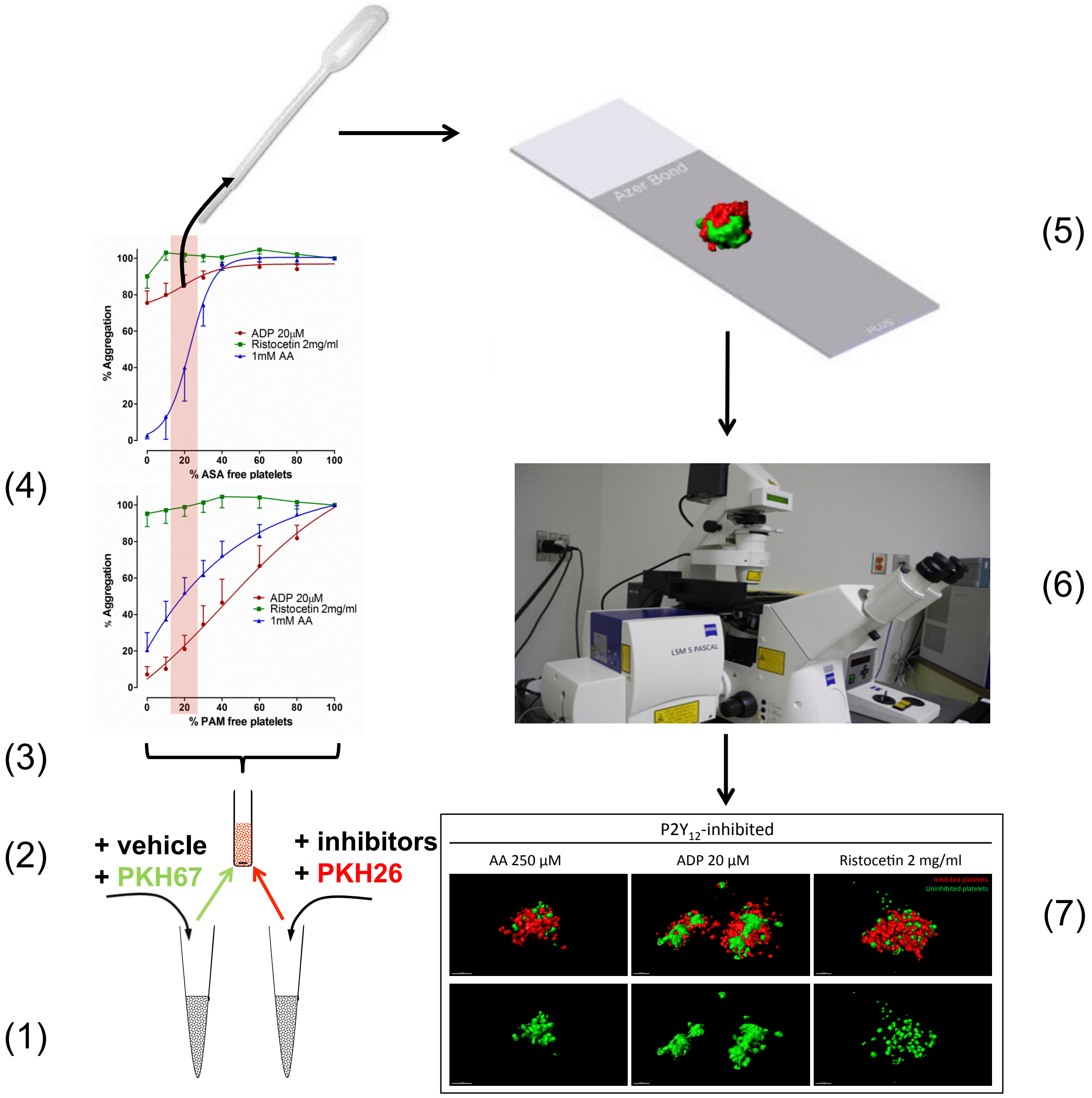

Figure SI: Schematic diagram summarising the experimental setup used for imaging platelet aggregates. 1 - platelet suspensions obtained from PRP; 2 – platelets labelled and treated with anti-platelet drugs; 3 – differently treated and labelled platelets combined in rising proportions; 4 – aggregation stimulated by a range of agonists; 5 – formed aggregates transferred to a microscope slide; 6 – aggregates imaged by confocal microscopy; 7 – images processed and analysed by Imaris software.

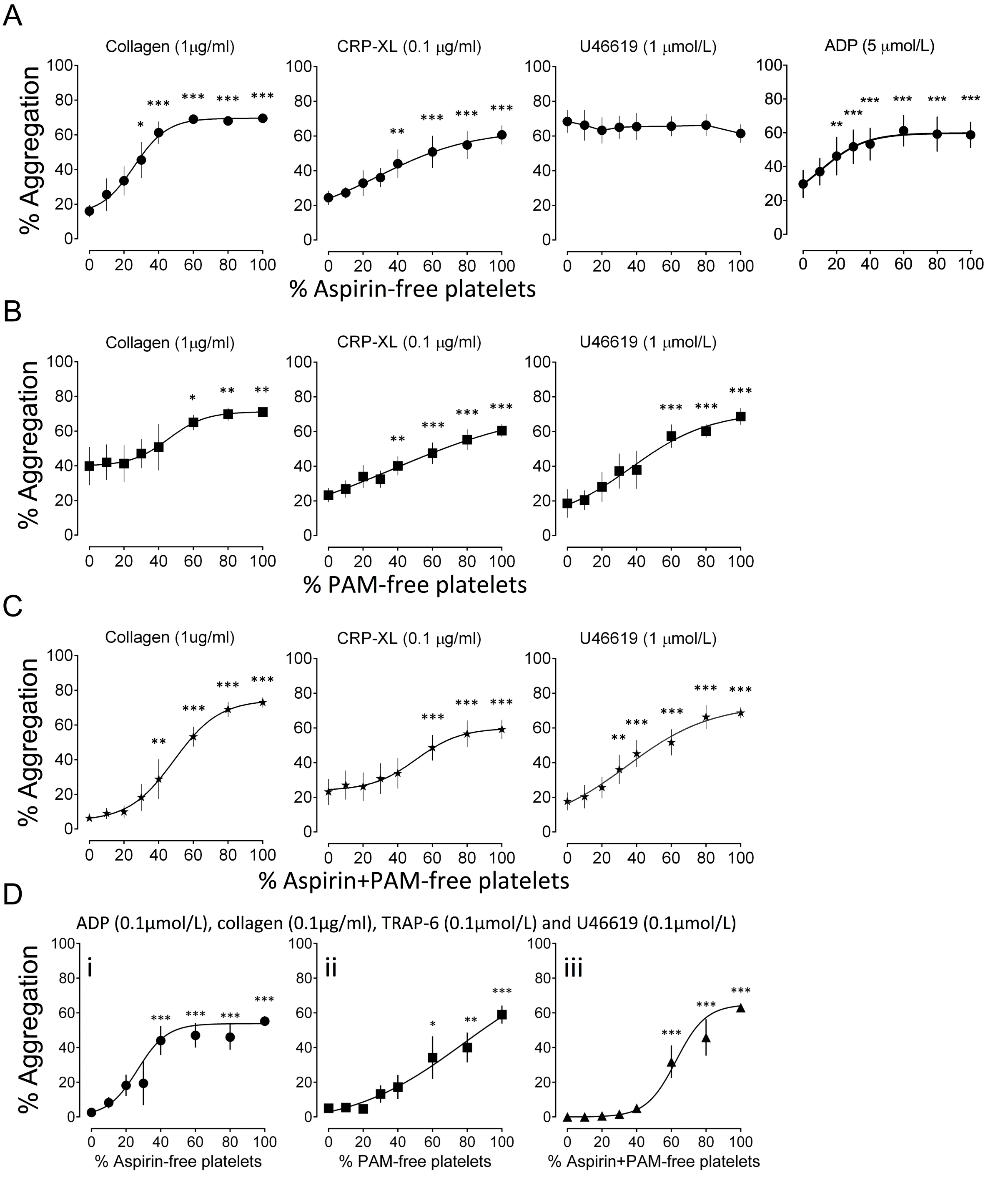

Figure SII: Light transmission aggregation in inhibited PRP containing rising proportions of drug-free platelets. Aggregatory responses to collagen (1 μg/ml), CRP-XL (0.1 μg/ml), U46619 (1 μmol/L) or ADP (5 μmol/L) after adding drug-free PRP to PRP inhibited with (A) aspirin (30 μmol/L), (B) PAM (3 μmol/L) and (C) aspirin+PAM. (D) The aggregatory responses to the combination of ADP (0.1 μmol/L), collagen (0.1 μg/ml), TRAP-6 (0.1 μmol/L) and U46619 (0.1 μmol/L) in drug-free PRP with (Di) aspirin, (Dii) PAM or (Diii) aspirin+PAM-inhibited PRP. Curves were constructed from multiple aggregatory responses using final aggregation values obtained by traditional LTA after five minutes aggregation. Percentage aggregation values are presented as mean±SEM of experiments using platelets prepared from 5-8 individuals. *** $p<0.001$, ** $p<0.01$ and * $p<0.05$ difference by paired ANOVA in aggregation from 100% drug-free platelets.

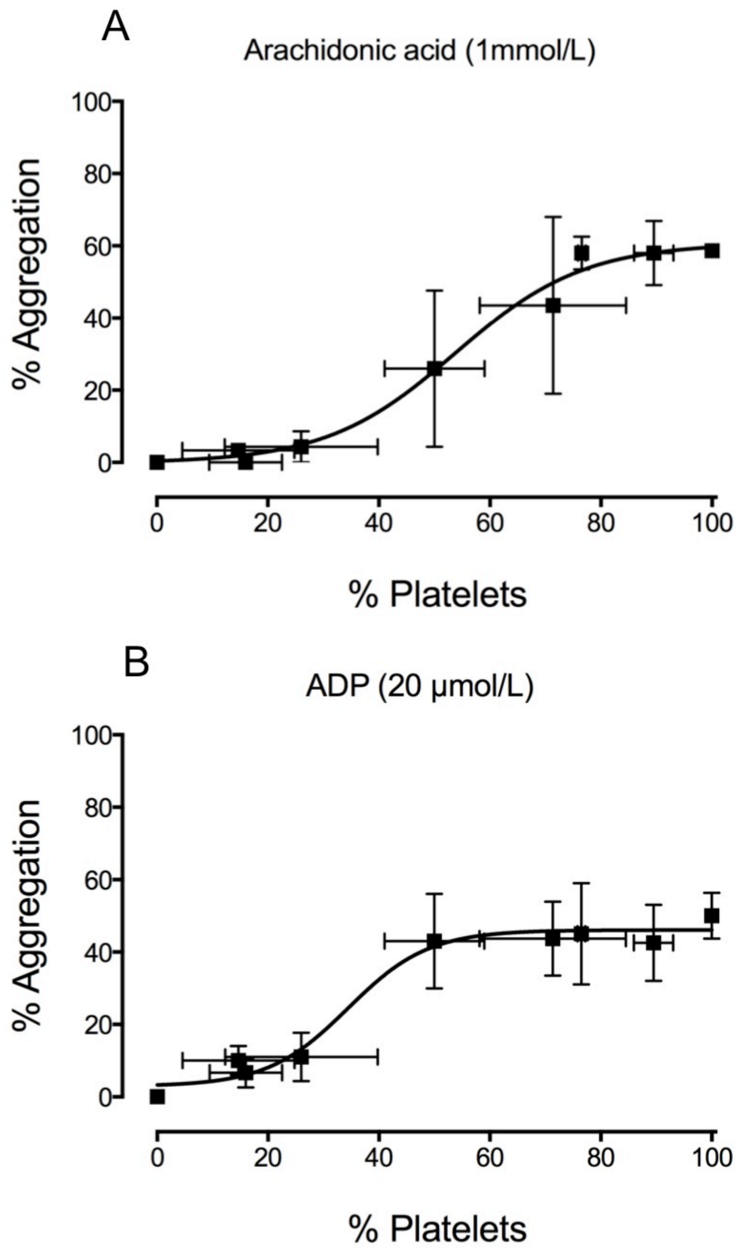

Figure SIII: Light transmission aggregation in PRP containing variable platelet concentrations. Effects of variable platelet concentrations, obtained by centrifuging whole blood at different speeds, on aggregatory responses to A) AA (1 mmol/L), or B) ADP (20 µmol/L). Data is presented as percentage of platelets obtained after centrifugation at 175 x g. Curves were constructed from multiple aggregatory responses using final aggregation values obtained by traditional LTA after five minutes aggregation. Percentage aggregation values and % platelets are presented as mean±SEM of experiments using platelets prepared from 3 individuals.

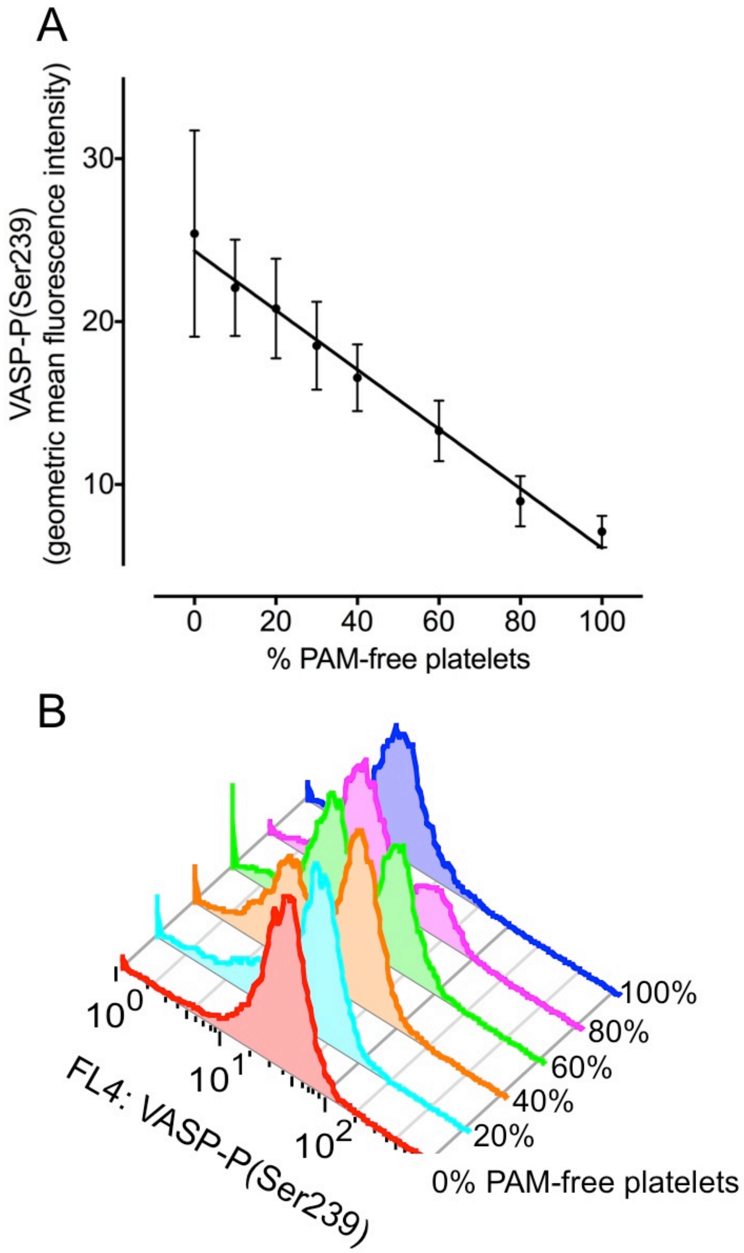

Figure SIV: Phosphorylation of vasodilator stimulated phosphoprotein (VASP) in PRP containing rising proportions of drug-free platelets. (A) Geometric mean fluorescence intensity of phospho(Ser^{239})-VASP, measured by flow cytometry after adding drug-free PRP to PRP inhibited with (PAM (3 μmol/L) and stimulated with ADP (20 μmol/L). B) Representative phospho(Ser^{239})-VASP fluorescence histograms of each PRP-proportion stimulated with ADP (20 μmol/L). Data are presented as mean±SEM of experiments using platelets prepared from 4 individuals.
